# Expectations Questionnaire for Adults with Cochlear Implants (EQA-CI): Translation, adaptation and cross-cultural validation to Brazilian Portuguese

**DOI:** 10.1016/j.bjorl.2025.101717

**Published:** 2025-09-26

**Authors:** Ingrid Barros Da Silva Santana, Fernanda Ferreira Caldas, Carolina Costa Cardoso, Francisco Wallison Lucena da Silva, Rayssa Pacheco Brito Dourado, Fayez Bahmad

**Affiliations:** a Universidade de Brasília (Unb), Brasilia, DF, Brazil; b Instituto Brasiliense de Otorrinolaringologia (IBO), Brasília, DF, Brazil

**Keywords:** Cochlear implantation, Treatment expectations, Adults, Translations, Surveys

## Abstract

**Objective:**

To translate, culturally adapt and validate the *Cuestionário de Expectativas* (CEA) for the Brazilian context, with focus on assessing the expectations of patients during pre-surgical stage.

**Methods:**

An analytical and descriptive study was carried out, including translation, back-translation, adaptation and validation of the CEA. The process involved 119 participants who answered the questionnaire, allowing the analysis of understanding of the items using the Content Validity Index (CVI) and Exploratory Factor Analysis (EFA). Reliability was assessed with Cronbach's Alpha, McDonald's Omega and Composite Reliability coefficients, using Item Response Theory (IRT).

**Results:**

The questionnaire had a total CVI of 0.974, indicating good comprehension by the participants, as well as high values for three internal consistency coefficients (⍺ = 0.828; ω = 0.854; CC = 0.900). The instrument showed great consistency indices and validity measures to be applied on a large scale.

**Conclusion:**

The validity and reliability of the instrument were confirmed. Therefore, the instrument was translated, adapted and validated for Brazilian Portuguese.

**Levels of evidence:**

Step 2.

## Highlights

• The QEA-IC can be utilized with accuracy and reliability.
• It identifies difficulties that can be adjusted to enhance outcomes.
• Understanding expectations enhances the performance of the cochlear implant.

## Introduction

Hearing loss impacts an individual's quality of life, limiting their participation in everyday situations.^1^ Untreated hearing loss can lead to high rates of depression, accelerated cognitive decline, and increased social isolation, in addition to generating significant economic burdens on society.^2^ Therefore, promoting hearing rehabilitation is essential.

Hearing rehabilitation is a broad concept encompassing a range of interventions, including the use of hearing devices such as Cochlear Implants (CIs), Hearing Aids (HAs), and Bone-Anchored Hearing Aids (BAHAs), supported by counseling and auditory perception training.

CIs are electronic devices that provide peripheral stimulation, enabling users to access speech sounds and improve their quality of life.^3^ However, not all CI users achieve the expected levels of speech recognition, whether in quiet or noisy environments. Moreover, conventional audiological assessments often fail to capture crucial aspects for adult CI candidates, such as reduced listening effort, enhanced social skills, and improved emotional well-being.^4^

According to Lilg et al.,^5^ the CI decision-making process requires managing expectations and clarifying aspects related to the technology, training, and surgical procedure. McRackan^6^ emphasizes that inadequate pre-surgical preparation may hinder an individual's adaptation to the new auditory experience, compromising their satisfaction with device use. Therefore, incorporating an expectations questionnaire into the CI surgical decision-making process can influence postoperative outcomes and enhance quality of life.^4^^,^^7^

Despite the importance of this topic, no questionnaires assessing the expectations of adult candidates for cochlear implant surgery have been published in Brazil. In recent literature, only a few studies address this issue, such as research focused on expectations among teenagers^8^ and investigations into parental perspectives on their children's cochlear implants.^9^ In this context, the Cuestionario de Expectativas para Adultos (CEA) is a valuable instrument, as it encompasses domains related to device usage, adaptation to new auditory stimuli, hearing practice, speech understanding, communication in noisy environments, and participation in social activities.^10^

Furthermore, there is a lack of internationally developed instruments validated for the Brazilian context. When such instruments do exist, they are often only partially adapted. It is necessary to systematize the methodological process in accordance with international guidelines to ensure a proper assessment of the test's psychometric properties.^11^

Thus, the CEA enables professionals to engage in discussions regarding CI expectations based on patients' responses. Therefore, this study aims to translate, culturally adapt, and validate this instrument for use in Brazil.

## Methods

This is an analytical, descriptive, and prospective study. The research was approved by the Research Ethics Committee of the Faculty of Health Sciences, under protocol number 6.766.359. It was conducted in the city of Brasília – DF, with participants providing informed consent by signing the Free and Informed Consent Form.

The study consisted of translating and adapting a questionnaire designed for the adult population, originally developed by Med-EL® and available in Spanish and English. The questionnaire comprises 16 items, each with five response options: True, Very Likely, Possibly, Not Very Likely, and False.

Recruitment was conducted in two phases. The first phase, focused on instrument adaptation, involved 19 adult cochlear implant candidates from an auditory rehabilitation center. Data were collected in person under the supervision of a qualified professional. The second phase, aimed at validation, included 100 participants recruited via the social media platforms Instagram and WhatsApp. These participants were adults residing in Brazilian states with reference centers for cochlear implantation. The final version of the questionnaire was made available in both paper and electronic formats (Google Forms).

Before completing the expectations questionnaire, participants underwent a brief interview to collect demographic and clinical data, including age, sex, level of education, laterality of hearing loss (bilateral or unilateral), and prior use of hearing aids. The duration of hearing loss could not be reliably determined for most participants.

A total of 119 adults participated in the study, with a mean age of 46.6 years. The sample consisted of 64 women and 55 men; 36 individuals had pre-lingual hearing loss, and 83 had post-lingual hearing loss. All participants presented with severe and/or profound sensorineural hearing loss in at least one ear. Regarding laterality, 95% had bilateral hearing loss, while 5% had unilateral loss. The majority (87%) reported prior use of hearing aids, whereas 13% did not.

Regarding educational attainment, 4% of participants had completed elementary school, 14% had completed high school, 60% held an undergraduate degree, and 22% had a postgraduate qualification.

Inclusion criteria were age 18 years or older, diagnosis of severe and/or profound sensorineural hearing loss in at least one ear, candidacy for cochlear implant surgery, Brazilian nationality, and literacy in Portuguese. The only exclusion criterion was refusal to participate.

## Translation, cultural adaptation, and validation stages

The translation, cultural adaptation, and validation of the *Cuestionario de Expectativas para Adultos* (CEA) into Brazilian Portuguese were authorized by the original authors of the instrument.

The translation and adaptation process followed the protocol established by Guillemin et al. (1993),^12^ and the validation process was conducted in accordance with the principles outlined in the Standards for Educational and Psychological Testing (SEPT).^13^ The translation methodology was carried out in six stages: I) Translation; II) Back-translation; III) Expert committee review; IV) Pre-test; V) Cultural adaptation; VI) Validation.

## 1^st^ Stage ‒ Translation

The original Spanish version of the questionnaire was independently translated into Brazilian Portuguese by two certified teachers of the Spanish language, working blindly to ensure individual perspectives. These versions were referred to as Translator-1 (T1) and Translator-2 (T2). The two translations were then compared, analyzed, and synthesized by the lead author to identify and resolve semantic discrepancies.

## 2^nd^ Stage ‒ Back-translation

A single back-translation of the synthesized version was carried out by two professional translators whose native language was Spanish and who were fluent in Portuguese. This step aimed to assess the semantic equivalence of the translated content. Importantly, the translators had no prior knowledge of the original questionnaire to avoid bias.

## 3^rd^ Stage ‒ Expert committee review

A multidisciplinary expert committee was formed, consisting of three Brazilian speech-language pathologists fluent in Spanish. After analyzing all versions of the questionnaire, they proposed modifications to certain terms to enhance clarity and cultural relevance for the Brazilian population. These changes are detailed in Table 2.

**Table 2:** Agreement percent.

	CVI	Interpretation	% Agreement
Q1	1.000	Acceptable	100.00%
Q2	1.000	Acceptable	100.00%
Q3	1.000	Acceptable	100.00%
Q4	0.895	Acceptable	89.47%
Q5	1.000	Acceptable	100.00%
Q6	0.947	Acceptable	94.74%
Q7	0.947	Acceptable	94.74%
Q8	0.947	Acceptable	94.74%
Q9	1.000	Acceptable	100.00%
Q10	1.000	Acceptable	100.00%
Q11	0.947	Acceptable	94.74%
Q12	0.842	Acceptable	84.21%
Q13	0.684	Unacceptable	68.42%
Q14	0.895	Acceptable	89.47%
Q15	0.947	Acceptable	94.74%
Q16	0.947	Acceptable	94.74%

Q, Questions; CVI, Content Validity Index.

## 4^th^ Stage ‒ Pre-test

The pre-test phase involved administering the questionnaire to 19 individuals from the target population to assess the intelligibility of the items. To evaluate whether the items were understood, two meta-questions were added after each item: 1) Was this item not understood? 2) Do you have any suggestions to make the test easier to understand?

## 5^th^ Stage ‒ Cultural adaptation

The objective of cultural adaptation was to ensure equivalence between the original instrument and the translated version, considering the lifestyle and cultural context of the target population. The Content Validity Index (CVI) was used as a quantitative measure of agreement among experts.^14^ Item Q13 presented the lowest agreement (CVI = 68.42%) and was therefore reformulated by the expert committee. Two versions of the item (Form A and Form B) were then presented to participants to determine whether the lack of comprehension was related to the type of hearing loss (unilateral or bilateral).

## 6^th^ Stage ‒ Validation

To validate the expectations questionnaire for adults using cochlear implants, psychometric evaluations were conducted. The validation process included collecting evidence of content validity, internal consistency, and construct validity, in line with internationally accepted standards for psychological and educational testing.

## Data analysis procedures

### Validity based on test content

The minimum sample size required for analyses using the Structural Equation Modeling (SEM) technique was calculated based on the ratio of 5–10 participants per item, resulting in a minimum required sample of 100 participants.^15^

Item-level parameters were analyzed using Item Response Theory (IRT), focusing on two key parameters: discrimination (a) and difficulty (b). The discrimination parameter reflects an item's ability to distinguish between individuals with different levels of the latent trait (θ). Its interpretation is categorized as follows: very low (0.01–0.34), low (0.35–0.64), moderate (0.65–1.34), high (1.35–1.69), and very high (≥1.70). The difficulty parameter refers to the level of the latent trait at which a respondent has a 50% probability of selecting a particular response category over another, and its values typically range from −3 to +3.^16^

### Validity based on internal consistency

Test reliability was evaluated through the internal consistency of latent factors. This was measured using Cronbach’s alpha (⍺), McDonald’s omega (ω), and Composite Reliability (CR) coefficients. In this study, all indicators were considered acceptable when values were greater than 0.70.^17^

### Construct validity

To assess construct validity, psychometric analyses were conducted to determine sensitivity and specificity. Item Characteristic Curves (ICCs) and Test Information Curves (TICs) were examined. ICCs help identify items with higher discriminatory power based on the steepness of the curve, while TICs indicate the overall amount of information provided by the test and the associated standard error of measurement across different levels of the latent trait.

## Results

The translations by T1 and T2 provided semantically equivalent sentences; however, some terms were modified to combine both versions and produce a final version that favored clarity and meaning.

Chart 1 presents the adaptations made to the questionnaire items in the final version.

**Chart 1:** Adaptation of items with discrepancies between translators.

Item	Original	Translation 1 (T1)	Translation 2 (T2)	Final version
Q2	Tomarme mi tiempo para adaptarme a un sonido nuevo y diferente	Demorar muito tempo para me acostumar a um som novo e diferente	Aceitar que necesito um tempo para me adaptar a um som novo e diferente	Precisar de um tempo para me adaptar a um som novo e diferente
Q3	Necesitar práctica auditiva con el equipamiento	Precisar de reabilitação auditiva com o implante	Necessitar prática auditiva com equipamento	Necessitar de terapia auditiva com o dispositivo auditivo
Q6	Tener un habla clara y fácil de entender	Ter uma fala clara e fácil de entender pelos outros	Ter um discurso claro e fácil de entender	Ter uma fala clara e fácil de entender pelos outros
Q10	Seguir una conversación con un grupo de personas	Acompanhar uma conversa com um grupo de pessoas	Seguir uma conversa com um grupo de pessoas	Acompanhar uma conversa com um grupo de pessoas
Q12	Continuar utilizando una ayuda de asistencia auditiva	Continuar utilizando de uma assistência auditiva	Ter que usar um dispositivo auditivo para ouvir.	Manter a assistência auditiva ao dispositivo, com acompanhamento de profissional especializado para programação e manutenção
Q14	Disfrutar de la música	Aproveitar uma música	Curtir a música	Apreciar a música

Q2, Question 2; Q3, Question 3; Q6, Question 6; Q10, Question 10; Q12, Question 12; Q13, Question 13.

In Stage II, the final version of the questionnaire was submitted to back-translation. The back-translators, who were native Spanish speakers fluent in Portuguese, confirmed that the back-translated version was semantically equivalent to the original Spanish version.

In Stage III, the expert committee reviewed all versions and confirmed the equivalence of the items.

In Stage IV, a pre-test was conducted with 19 participants. The Content Validity Index (CVI) was used to assess the participants’ understanding of each item.

Results indicated that six items (Q1, Q2, Q3, Q5, Q9, Q10) showed 100% agreement. Questions Q6, Q7, Q8, Q11, Q15, and Q16 achieved 94.74% agreement. Items Q4 and Q14 had 89.47%, and Q12 reached 84.21%. Item Q13 was the only one that fell below the acceptable threshold, with an agreement rate of 68.42%, indicating participants had difficulty understanding this question.

Based on these results, item Q13 was revised to improve clarity and ensure content validity. Given the diagnostic variability of participants (unilateral vs. bilateral hearing loss), two versions of the questionnaire were created ‒ Form A and Form B ‒ differing only in the phrasing of item Q13 (Table 1).

**Table 1:** Adaptation of question 13 into Brazilian Portuguese.

Steps	
Original	Continuar utilizando dispositivo auditivo en el otro oido
Translate	Continuar usando o dispositivo auditivo na outra orelha
Back-translation	Continuar usando el dispositivo auditivo en el otro oído
Adaptation after A and B forms	Necessitar do dispositivo auditivo na outra orelha para escutar melhor, se tiver perda auditiva bilateral

After the revision, item Q13 was reassessed using the CVI and reached 100% agreement. Overall, the instrument demonstrated excellent content validity, with an average CVI of 0.974 (Table 2).

Therefore, a psychometric analysis gathered evidence of content validity based on the understanding of items by the target population and could be applied on a large scale.

### Validation

Analytical assumptions confirmed the factorability of the data matrix [KMO = 0.735, Bartlett’s Test (15) = 92.705, p < 0.001]. The Hull method indicated the presence of a single latent factor (CFI_Hull = 0.968). Based on this, Exploratory Factor Analysis (EFA) was performed, showing an acceptable model fit: χ^2^(104) = 264.887, χ^2^/df = 2.547, CFI = 0.938, TLI = 0.928, SRMR = 0.138, RMSEA = 0.125 (90% CI 0.106–0.144).

However, residual indices were considered excessively high.

The model’s factor loadings (Table 3) showed that items Q2, Q3, and Q13 had loadings below the acceptable threshold (0.30) for representing a latent factor. As a result, these items were removed from subsequent internal consistency calculations. After their exclusion, the remaining items demonstrated excellent internal consistency, indicating a strong correlation among the questions.

**Table 3:** Validity evidence based on internal structure.

Items	λ	a	b1	b2	b3	b4	S_χ^2^	RMSEA
Q1	0.444^a^	0.789	−4.778	−4.096	−2.977	−1.920	10.211	0.068
Q2	**0.183**	**0.381**	−12.277	−7.976	−4.971	−2.461	22.556	0.079
Q3	**0.144**	**0.250**	−14.050	−8.058	−4.656	NA	9.821	0.000
Q4	0.480^a^	0.850	−5.808	−4.985	−2.325	−1.033	13.018	0.004
Q5	0.719^a^	2.222	−2.586	−1.983	−0.722	0.256	11.308	0.000
Q6	0.701^a^	1.485	−3.816	−2.688	−1.713	−0.338	15.691	0.076
Q7	0.702^a^	1.777	−2.561	−2.395	−1.128	−0.207	20.711	0.062
Q8	0.705^a^	1.984	−2.864	−1.795	−0.743	0.082	21.996	0.062
Q9	0.838^a^	2.884	−2.114	−1.381	−0.366	0.476	18.820	0.067
Q10	0.888^a^	3.863	−2.338	−1.458	−0.537	0.367	**32.102**^a^	**0.122**
Q11	0.528^a^	1.103	−4.692	−1.909	−0.570	NA	17.687	0.060
Q12	0.461^a^	0.705	−5.273	−3.990	−3.415	−1.992	17.533	**0.123**
Q13	**0.240**	**0.371**	−5.623	−4.159	−2.745	−1.327	11.064	0.008
Q14	0.674^a^	1.195	−3.467	−3.195	−1.376	−0.266	19.934	0.033
Q15	0.613^a^	1.010	−3.921	−2.943	−1.753	−0.349	16.107	0.000
Q16	0.495^a^	0.979	−2.029	−1.086	0.140	1.552	20.755	0.000
⍺	0.828							
ω	0.854							
CC	0.900							

Guttman Lambda (λ): varies from 0 to 1, with a value of 0.3 or more required to show strong predictability.

Discrimination of item (a): being a value considered very low (0.01–0.34), low (0.35–0.64), moderate (0.65–1.34), high (1.35–1.69) and very high (above 1.70).

Difficulty of item (b): being a strong value if −3 < b > +3.

RMSEA: evaluates whether the model fits the population. Values close to zero suggest a well-adjusted model.

Cronbach's alpha coefficient (⍺), McDonald's Omega (ω) and Composite Reliability (C.C.): evaluate internal consistency, considering values above 0.70 to be adequate.

a p < 0.05; bold: unsatisfactory values.

**Fig. 1:**
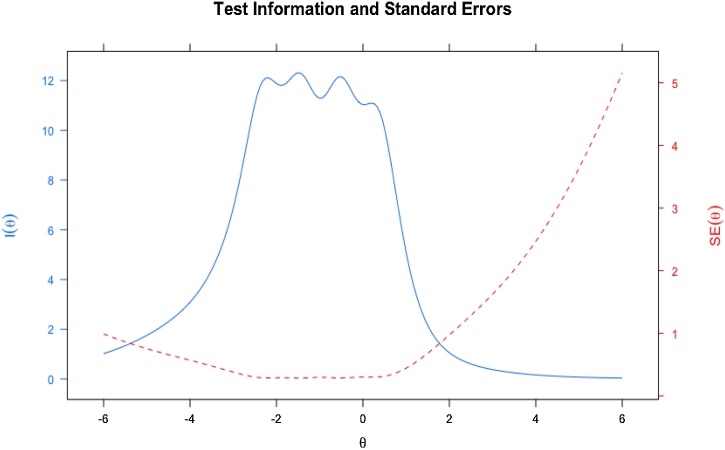
Test Information Curve (TIC) related to the sensitivity of the questionnaire.

**Fig. 2:**
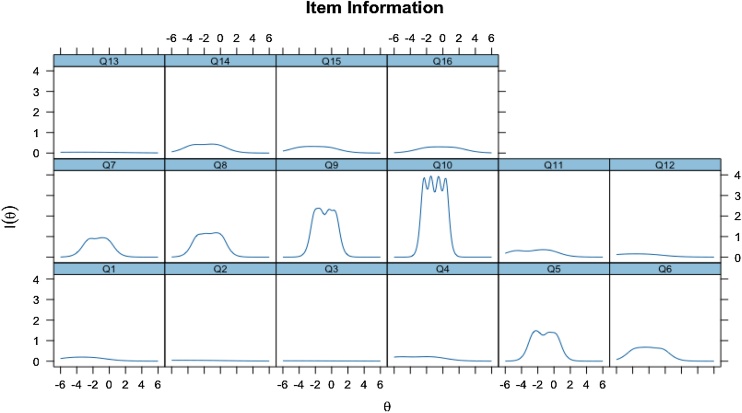
Item Characteristic Curves (ICCs) related to the information collected from each question.

The Item Characteristics Curve (ICC) was analyzed, and the result is demonstrated in Fig. 1. Results presented in sinuous waves of the ICCs indicate that items Q10, Q9, and Q5 presented the largest amount of measured information among the others, since the waves present larger curves, while items Q2, Q3, and Q13 presented the smallest amount of measured information. Fig. 2 demonstrates the analysis of the Test Information Curve (TIC), which is responsible for verifying whether the items are sensitive to expectation or not. TIC indicated that the instrument's information peak accurately measures the lowest levels of expectation.

Discrimination scores on this instrument assessed whether the individual will be sensitive to expectations, and the difficulty measured how much expectation an individual needs to score. Items Q2 and Q3 showed low discrimination indicators (α < 0.30), suggesting they were unable to adequately differentiate individuals with distinct levels of expectation regarding the cochlear implant, possibly due to the similarity in responses given, regardless of the participant's profile.

The questionnaire demonstrated good reliability, sensitivity to measure expectations, and strong evidence of validity for use in Brazilian Portuguese. The final version of the translated and adapted instrument is presented in Chart 2.

**Chart 2:** Final Brazilian Portuguese version of the questionnaire.

Questionário de Expectativas de Adultos para o Implante Coclear (QEA-IC)
Nome:
Idade:____________anos Data de Nascimento: _____/_____/_____
Escolaridade:
Tem perda bilateral: Implante Coclear indicado para:
() Sim () Não () OE () OD
Você usa aparelho auditivo (AASI) na outra orelha: () Sim () Não
Como responder: **Circule a alternativa que atende suas expectativas**
Eu vou…
**1. Precisar colocar o processador externo durante todas as horas do dia em que estiver acordado**
Por favor, circule: Verdadeiro Muito Provável Possivelmente Improvável Falso
**2. Precisar de um tempo para me adaptar a um som novo e diferente**
Por favor, circule: Verdadeiro Falso
**3. Necessitar de terapia auditiva com dispositivo auditivo**
Por favor, circule: Verdadeiro Falso
**4. Ouvir mais sons ambientais**
Por favor, circule: Verdadeiro Muito Provável Possivelmente Improvável Falso
**5. Ser capaz de identificar de onde vêm os sons**
Por favor, circule: Verdadeiro Muito Provável Possivelmente Improvável Falso
**6. Ter um fala clara e fácil de entender**
Por favor, circule: Verdadeiro Muito Provável Possivelmente Improvável Falso
**7. Melhorar minhas habilidades de comunicação ao nível dos meus colegas ouvintes**
Por favor, circule: Verdadeiro Muito Provável Possivelmente Improvável Falso
**8. Entender o que as pessoas dizem sem leitura labial**
Por favor, circule: Verdadeiro Muito Provável Possivelmente Improvável Falso
**9. Entender as pessoas ao meu redor quando houver ruído de fundo**
Por favor, circule: Verdadeiro Muito Provável Possivelmente Improvável Falso
**10. Acompanhar uma conversa com um grupo de pessoas**
Por favor, circule: Verdadeiro Muito Provável Possivelmente Improvável Falso
**11. Melhorar em meus estudos/trabalho**
Por favor, circule: Verdadeiro Muito Provável Possivelmente Improvável Falso
**12. Manter a assistência auditiva ao dispositivo, com acompanhamento de profissional especializado para programação e manutenção**
Por favor, circule: Verdadeiro Muito Provável Possivelmente Improvável Falso
**13. Necessitar de dispositivo auditivo na outra orelha para escutar melhor, se tiver perda auditiva bilateral**
Por favor, circule: Verdadeiro Muito Provável Possivelmente Improvável Falso Não se aplica
**14. Apreciar a música**
Por favor, circule: Verdadeiro Muito Provável Possivelmente Improvável Falso
**15. Melhorar o uso do telefone**
Por favor, circule: Verdadeiro Muito Provável Possivelmente Improvável Falso
**16. Ter audição normal**
Por favor, circule: Verdadeiro Muito Provável Possivelmente Improvável Falso

## Discussion

The role of patient expectations has been extensively studied across various medical disciplines; however, it remains underexplored in the context of cochlear implantation.^18^ In audiological practice, particularly during discussions and preoperative counseling, professionals often rely solely on speech recognition test results. Nevertheless, this approach is insufficient, as it overlooks the complex social, communicative, and emotional dimensions of the individual.^6^

The use of a standardized questionnaire to assess expectations regarding Cochlear Implant (CI) outcomes enables a more comprehensive understanding of the candidate profile. The Expectations Questionnaire for Adults with Cochlear Implants (EQA-CI) can be integrated into clinical routines to align expectations, support informed decision-making, and promote realistic goal setting. Furthermore, it aids in understanding the actual benefits of cochlear implantation ‒ such as auditory adaptation and consistent device use ‒ while identifying and addressing misconceptions or unrealistic expectations.^10^

Research has shown that patient expectations are multifaceted, involving functional, psychosocial, emotional, and device-related dimensions. Functionally, many candidates anticipate significant improvements in speech comprehension (especially in quiet environments), greater ease using the telephone, and reduced listening effort.^4^, ^5^, ^19^

On the psychosocial level, patients often expect to resume social activities, increase their independence, and enhance their quality of life. Emotionally, expectations are frequently centered on overcoming frustration, isolation, and anxiety related to hearing loss, as well as regaining self-confidence and a sense of social belonging.

With regard to the device itself, candidates commonly expect natural sound perception, ease of use, and enjoyment of music with a sound quality comparable to normal hearing.^4^, ^5^, ^19^ The expectations of CI candidates are, therefore, a critical component of the clinical process. If not properly managed, expectations can significantly impact treatment satisfaction, adherence to device use, and postoperative quality of life. Prentiss et al.^20^ emphasized that misaligned expectations ‒ whether overly optimistic or pessimistic ‒ may compromise perceived treatment success and, in some cases, may even constitute a contraindication for cochlear implantation.

Poorly calibrated expectations can lead to adverse outcomes such as dissatisfaction, frustration, and persistent social withdrawal, ultimately affecting the individual’s quality of life.^19^ They also increase the likelihood of device abandonment and treatment discontinuation, reducing the overall benefit of the intervention.^6^ Many patients expect the CI to restore “normal” hearing, enabling effortless speech comprehension in noisy environments and music appreciation; however, such outcomes are not always attainable, particularly depending on the etiology of the hearing loss.^5^

The EQA-CI was validated with a heterogeneous sample of 100 participants, allowing the collection of robust evidence for validity. This diversity supports the inference that participants’ responses were not influenced by demographic or clinical variables. McRackan et al.^4^ observed that variables such as age at surgery, duration of hearing loss, and whether the hearing loss was unilateral or bilateral did not significantly affect preoperative expectations in CI candidates.

In terms of individual items, the Content Validity Index (CVI) revealed that item Q13 was particularly challenging to understand, likely because not all CI candidates present with bilateral profound hearing loss. In some cases, bilateral implantation is contraindicated due to factors such as age, type and degree of hearing loss, auditory nerve integrity, or duration of auditory deprivation. As a result, item Q13 was revised for greater clarity, yet retained in the final version to ensure comprehensive coverage of expectations.

Items Q5, Q9, and Q10 elicited the highest levels of expectation. These items refer to sound localization, speech comprehension in noisy environments, and the ability to follow conversations, respectively. These findings are particularly relevant, as these auditory abilities are among the most impaired by hearing loss and are crucial for successful social interaction. Deficits in these domains affect processes such as binaural separation and auditory discrimination.^21^ Moreover, as noted by Illg et al.^5^ and McRackan et al.,^4^ many CI candidates harbor elevated expectations regarding the restoration of these capabilities ‒ expectations that may not always be fully met postoperatively.

Items Q2 (“needing time to adapt to a new and different sound”) and Q3 (“requiring auditory therapy with the hearing device”) were retained in the Brazilian Portuguese version. The use of a cochlear implant enhances speech perception and facilitates neural reorganization in response to novel auditory input.^22^ Furthermore, Cambridge et al.^23^ emphasized the importance of ongoing auditory training to support the adaptation process to electric hearing.

In conclusion, the convergence of psychometric findings confirms that the EQA-CI demonstrates strong precision, reliability, and validity for assessing expectations in the context of cochlear implantation in Brazil. As such, it is a valuable tool for evaluating patient expectations, informing clinical decisions, and optimizing treatment outcomes.

### Final considerations

One limitation of this study is the relatively small sample size, which was insufficient to establish normative scale scoring.^24^ Therefore, future research should aim to increase the sample size to enable the development of a quantitative scoring system for the questionnaire. Expanding the sample will also allow for more robust statistical analyses and broader generalizability of the results.

## Conclusion

The psychometric analyses including assessments of internal consistency, content validity, factor structure, and item response theory demonstrate that the questionnaire was successfully translated and culturally adapted for Brazilian Portuguese. The instrument presents strong validity and reliability indices, supporting its use for evaluating expectations in adult cochlear implant candidates within the Brazilian context.

## ORCID ID

Ingrid Barros Da Silva Santana: 0009-0002-1914-4082

## Financial disclosures

This research did not receive any specific grant from funding agencies in the public, commercial, or not-for-profit sectors.

## Declaration of competing interest

The authors declare no conflicts of interest.
